# Entropy as a Geometrical Source of Information in Biological Organizations

**DOI:** 10.3390/e24101390

**Published:** 2022-09-29

**Authors:** Juan Lopez-Sauceda, Philipp von Bülow, Carlos Ortega-Laurel, Francisco Perez-Martinez, Kalina Miranda-Perkins, José Gerardo Carrillo-González

**Affiliations:** 1 Consejo Nacional de Ciencia y Tecnología (CONACYT), Avenida Insurgentes Sur 1582, Colonia Crédito Constructor, Alcaldía Benito Juárez, Mexico City 03940, Mexico; 2 Departamento de Procesos Productivos, Universidad Autónoma Metropolitana, Avenida de las Garzas No. 10, Colonia El Panteón, Lerma de Villada 52005, Mexico; 3 Departamento de Sistemas de Información y Comunicaciones, Universidad Autónoma Metropolitana, Avenida de las Garzas No. 10, Colonia El Panteón, Lerma de Villada 52005, Mexico; 4 Secretaría de Medio Ambiente y Recursos Naturales (SEMARNAT), Avenida Ejército Nacional 223, Colonia Anáhuac, Alcaldía Miguel Hidalgo, Mexico City 11320, Mexico

**Keywords:** differential entropy, discrete entropy, geometrical information, heterogeneity, information theory

## Abstract

Considering both biological and non-biological polygonal shape organizations, in this paper we introduce a quantitative method which is able to determine informational entropy as spatial differences between heterogeneity of internal areas from simulation and experimental samples. According to these data (i.e., heterogeneity), we are able to establish levels of informational entropy using statistical insights of spatial orders using discrete and continuous values. Given a particular state of entropy, we establish levels of information as a novel approach which can unveil general principles of biological organization. Thirty-five geometric aggregates are tested (biological, non-biological, and polygonal simulations) in order to obtain the theoretical and experimental results of their spatial heterogeneity. Geometrical aggregates (meshes) include a spectrum of organizations ranging from cell meshes to ecological patterns. Experimental results for discrete entropy using a bin width of 0.5 show that a particular range of informational entropy (0.08 to 0.27 bits) is intrinsically associated with low rates of heterogeneity, which indicates a high degree of uncertainty in finding non-homogeneous configurations. In contrast, differential entropy (continuous) results reflect negative entropy within a particular range (−0.4 to −0.9) for all bin widths. We conclude that the differential entropy of geometrical organizations is an important source of neglected information in biological systems.

## 1. Introduction

In the context of shapes and forms in biology, there has been an historical effort to find the source of some patterns and the fundamental nature of their seemingly steady basic arrangement. Traditionally, the bottom-up logic of biological developed structures as dynamical time-space expression processes has been extensively approached either by Neo-Darwinism (e.g., genetic blueprint or functional viewpoints) or by biological structuralism (e.g., fractal biologic patterns from chaos theory). In fact, these approaches are supported by epistemological conceptions defining traditions of research work, such as positioning whole organisms as being made of atomic and separate parts (i.e., systematics) or the holistic dynamical system approach of the structuralist point of view (e.g., Turing patterns). In contrast, our perspective employs Shannon entropy to understand biological organizations as a geometric whole whose configurations defining their steady state result from an inherent and specific level of information. One important example of steady states is derived from a prevailing and well stereotyped distribution of cellular polygons in metazoans tissues (epithelium). The question concerning whether patterns and shapes are an active source of geometrical information, stability, and variability during developmental processes and evolution represents an intriguing issue that requires further study. Although that “geometrical information” sounds very similar to the interdisciplinary field of Information geometry is important to say that they are different approaches with some important convergences that will be treated briefly at discussion.

In this work, the geometric properties of individual discrete elements in forms are not simple intrinsic features biologically exposed as outcomes. Instead, we understand them as both independent spaces in a larger whole and as units defining interacting properties inside of a larger whole of geometric information. In this line of reasoning, our main methodological question about shapes arises: Is there a way to quantify geometrical order in biological organizations using levels of information? This question has been tackled from other perspectives. There is an important amount of work related to quantifying information at different complexity levels in biological networks [1,2,3,4], ecosystems [5,6,7,8,9], molecular entropy [10], and cellular entropy [11], to name a few approaches. Furthermore, the characterization of ecological landscape heterogeneity (e.g., urban, sociological, and economical properties at multiple scales associated with them) have been approached [12,13,14] using spatial entropy and complexity tools. However, in the context of pure biology, the underlying informational order behind the geometry of general biological organizations is still not quite clear. However, there are some intuitions regarding quantitative values for biology and architecture [15]. Nevertheless, the limits defining quantitative parameters of order according to entropy, as a generic value for biological organizations, is still an issue which has yet to be solved. We maintain that an important aspect of our research is the selection of a set of biological forms to work on.

The core idea of measuring spatial heterogeneity to determine the geometrical entropy of a particular biological form is derived from a previous analysis of geometrical constrictions in five-fold morphologies (polygonal random disc organizations) [16]. In that work, it was found that spatial organization of five-fold morphologies is statistically lower than all other planar disc organizations (three to ten-fold morphologies) in terms of spatial heterogeneity (unequal distribution of space inside polygons). In fact, the authors found a statistical basis corresponding to the most frequent morphologies in biological disc organizations (three, four, five, and six disk partitions are typically found in flowers, fruits, and other biological organizations) [16]. While we found a statistical value to approach the key idea of low heterogeneity for related morphologies in nature, the authors were unable to capture quantitatively the geometrical limits of biological organizations within a formal framework of reference.

Much work has been carried out regarding the larger geometrical context of cells and the physical causalities of interactions into cell aggregates using meshes [17,18,19,20,21,22,23], which notably enforces our background. This work derives from physical parameters and describes geometrical properties while not strictly determining levels of geometric information. The characterization of ‘self-assembled 2D patterns with Voronoi entropy’ represents a certain approach for achieving geometry as a source of organization, employing levels of spatial heterogeneity at different scales [24,25]. However, the main results of this kind of work provide some insight about entropy in the context of matter organization and ecological dynamics, even stirring research on material sciences and cellular aspects (including topics such as the informational limits of generic order in biology). Living systems show an important reduction of entropy, reaching very low values along self-organization as an ostensibly consistent rule [1,3,6,10,13,16,24,25,26,27,28]. Such behavior has been associated with biological and physical constraints [29,30], with some proposals linking it to pure geometry [26,27,28,31,32]. The hypothesis we will try to verify in this work is as follows: the more self-organized a system, the less entropic is its behavior. Hence, we expect that this information is related with the ordering of geometric parts throughout biological structures. Accordingly, Shannon entropy shall indicate the amount of information considered (besides being a proxy of geometrical heterogeneity). In addition, Shannon entropy is the average of a variable’s uncertainty that reflects how much information is associated with the probability of a given event. In this paper, we propose that its range [i.e., heterogeneity, non-heterogeneity] can be translated into bits of information between 0 and 1.

The set of organizations that we choose is based on looking for strictly biological samples made of polygons at two size levels (cellular and ecological), simulations of biological samples, and experimental controls (random simulations and poisson tessellations). The main idea was to generate a proper collection of biological samples to detect particular levels of informational entropy using the unique simplicity of polygons as a general feature for a data source. Those polygons have levels of heterogeneity which will be our source of data used to establish levels of entropy in order to identify biological particularities.

To develop this idea, this paper is organized as follows. First, there is an exposition of the collecting method and features and categorization of biological images samples and non-biological mesh simulations in Section 2. These data will define the material to work on besides random polygons with different numbers of sides (Appendix A). The mathematical framework and the statistical motivation to work on these polygons and the main background used to define heterogeneity in spatial organization of polygonal shapes and meshes are given in the ‘Methods’ section. The procedure used to measure the quantity of information in geometrical meshes of biological and non-biological systems using Shannon entropy and the associated statistical distributions of internal partitioning in shapes is shown in Section 3. Finally, Section 4 and Section 5 correspond to discussion and conclusions, respectively.

## 2. Materials and Methods

### 2.1. Materials

The outline of an area or figure is a shape that can be a determined configuration of discrete elements, which sometimes can be understood as a population of geometric parts which serve as constitutive elements. Our approach here is to determine levels of geometric information using Shannon entropy as the main theoretical framework. Therefore, informational entropy would allow for the quantification of order and disorder levels from discrete and continuous geometric variables. Continuous approaches which are able to characterize chemical, physical, and biological patterns, based on the continuous measure of symmetry, were introduced [33,34,35,36,37,38]. Suitably, the first focus of our research is on extract basic discrete and continuous geometric principles of polygons immersed into larger whole organizations (called polygonally shaped patterns, or PSP) in order to standardize levels of biological information given several amounts of heterogeneity (i.e., unequal distribution of space inside a given area). Rather than just looking at polygons and their aggregates as mathematical outcomes derived from computing simulations, in this paper we developed a statistical process to detect levels of information from them. Our method points out to spatial heterogeneity of polygons as a free scale informational substrate that can be approached on a wide range of biological size scales (which also can be easily translated into an informational entropy metric description). Hence, the material of our work will be the areas (polygons) and their associated sub-areas (triangles) defining levels of heterogeneity. Our procedure satisfies the fact that we may work with sets of polygonal shapes as an informational substrate upon we can discern levels of geometrical heterogeneity getting a width spectrum of numerical data. This metric was tested into meshes (biological, non-biological, and random polygonal arrangements) and simulated random discs with different number of sides. Finally, we will retrieve the informational limits of biological structures whose geometry would potentially be biologically representative in terms of their closeness with nature images due to the informational entropy associated.

The first step was the establishment of a collection of biological images and sample data to work on. Several biological cell organizations have been used as models to define geometric parameters. In that sense, an important number of studies have analyzed the topological properties of many cell organizations [18,19,20,21,22,26,27,28,32,39,40,41,42,43,44]. Also, a lot of epithelium models have extensively used anatomical parts, developmental stages, and tissue variations images. In fact, there is a prevailing and well stereotyped distribution of cellular polygons (SDCP) conserved in proliferating metazoans tissues with a polygonal frequency of 29% of 5-sided polygons, 49% of 6-sided polygons, and 20% of 7-sided polygons [20,40,41]. In that context, some other images of biological cell organizations are available online, such as histological samples derived from different human tissues [20,42]. Currently, it is widely accepted that although variation in those organizations exists, there is just a narrow range of variations of cellular polygonal distributions [20,40]. In this regard, samples of polygonal meshes are directly comparable even if some of them are from different origin or scale due to all of them are PSP [45,46], including biological natural images, biological simulations, non-biological simulations (such as random meshes and Poisson-Voronoi tessellations), and random polygons. Therefore, levels of Shannon entropy in polygonal meshes and sets of random polygons turn into a window of universal and comparable information if we approach them from a pure geometric perspective.

#### Collecting Samples

**Figure 1:**
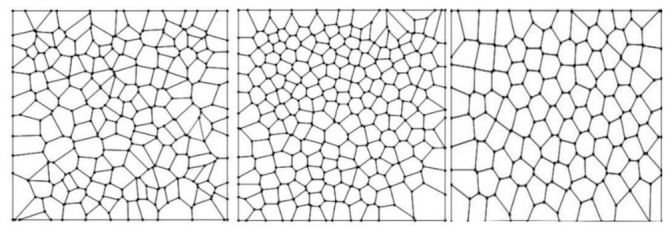
Three general types of schematic mosaics were analyzed. Left: random arrangements (RA). Center: natural images (BIO, schemes of aggregates of cells and ecological polygonal meshes) extracted from the web; muscular dystrophy (MD), drosophila prepupal wing discs (dWP), middle third instar wing discs (dWL), normal human biceps (BCA), pseudo-stratified drosophila wing disk epithelium (PSD), and ecological patterns (NFC and EOP). Right: processed non biological images (non BIO) extracted from the web which we named, control simulation (CS), simulation at equilibrium (SAE), atrophy simulation (AS), simulation out of equilibrium (SOE), and Poisson–Voronoi tessellation (PT).

We collected samples of images (online) looking for a broad and representative set of biological organizations in order to support our main hypothesis (i.e., that geometric information defined by the Shannon entropy of spatial polygonal heterogeneity is a proper parameter able to define the limits of a generic biological organizational value using PSP). Thus, the establishment of a measure of spatial organization able to determine the geometrical entropy of order for biological forms must be analyzed measuring biological and non-biological organizations (Figure 1). At the tissue level, we used images from proliferating drosophila prepupal wing discs (dWP) [20,41,43], middle third instar wing discs (dWL) [41,43], normal human biceps (BCA) [20], muscular dystrophy from skeletal muscles (MD) [42], and pseudo stratified drosophila wing disk epithelium (PSD) [40]. Also, at the ecological level polygonal meshes derived from Namibia fairy circles (ecological patterns associated with SDCP convergences) images were integrated into the analysis (NFC) and ecological oak patterns (EOP) [45,46,47,48]. The global tag to encompass MD, dWP, dWL, BCA, PSD, and NFC is called BIO. The non-biological meshes were different diagrams resulting from different vertex model simulations. Those simulations were based on quantified distances from SDCP, which is traditionally used as reference in epithelial studies [49,50,51]. The closeness with SDCP can be defined through an optimal paths approach using iterations of Lloyd’s algorithm and other cellular biophysical conditions in order to investigate the effects of cell divisions on topology [20]. In contrast, other work reached equilibrium states by seeking minimal potential energy [50]. Given this, there were epithelium simulations which we defined as control simulations (CS) [20,32], simulation out of equilibrium (SOE) [20,32], simulation at equilibrium (SAE) [20,32], atrophy simulation (AS) [20], and Poisson–Voronoi tessellation (PT) [20]. We consider CS, SOE, SAE, AS, and PT altogether as non-biological meshes (non BIO), since they were derived from algorithms and not from actual biological samples. In addition, in order to have a reference to contrast numerical values of nature typical arrangements we also include planar discrete areas (PDA; Section 2.2.1). Finally, we incorporated an algorithmic routine [45] to develop random arrangements (RA) into the global analysis as a control. Therefore, the analysis will include three PSP mesh categories, BIO, non BIO, RA, and data from PDA (Table 1; summary of category, abbreviation, name and number of samples).

### 2.2. Methods

#### 2.2.1. Mathematical Description of Shapes Γ and Heterogeneity of Spatial Organization

The establishment of a measure of heterogeneity able to determine the geometrical entropy of biological organizations is derived from a previous analysis of spatial constrictions in five-fold morphologies [16]. The algorithm to simulate partitions and shapes Γ-PDA (planar discrete areas inside a disc; Box 1) is extensively supported in Appendix A. Here, our main methodology goes beyond, focused on statistical measurements of geometrical heterogeneity onto biological and non-biological PSP, associating levels of entropy to them using fundamentals features of shapes Γ.

A former statistical analysis is derived from the study of partitions (areas) and their sub-localities (sub-areas) arising from computational constructions named Γ shapes. Generically, a shape Γ is a set of numerical values able to be analyzed statistically which is composed of sub-localities which are areas inside a partition $P_{i}$ (Box 1). Therefore, there are two particular cases of Γ shapes. Tthe first particular case of shape Γ can be a set of sub-areas derived from a partition $P_{i}$ being a disc simulation with a given number $N_{i}$ of sub-localities (Γ-PDA). The second one is a regular or irregular polygon with any number of sides. In that sense, each shape Γ can be achieved as a set of numerical sub-areas that can be subject to be statistically analyzed. The main idea used to establish the generic name of shape Γ is that it is useful to name either geometric objects (e.g., irregular and regular polygons or PDA) or areas (numeric values inside discs simulations or Γ-PDA) associated with either discs or any 2D simulated or not simulated polygonal shape derived from meshes.

Box 1 **Partition number.**

Figure a–c shows the process of partitioning using, as an example, five sub-localities. The concentric scheme at figure d shows three levels of variability (shadow zones limited by 1, 4, and 8) according to the scale given by the first circle radius. These shadow restricted zones are areas whose random points define sub-localities according to a particular partition number (figure a–c). This methodology is applied to partition number $P_{i}$ using discs with 3, 4, 5, 6, 7, 8, 9, and 10 sub-localities. The second concentric circle limits the variation of area once that Voronoi algorithm is running in order to limit as much as possible the area variability.

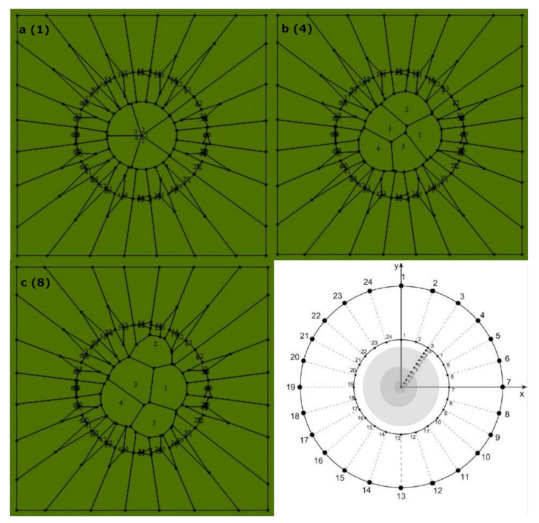

**Figure 2:**
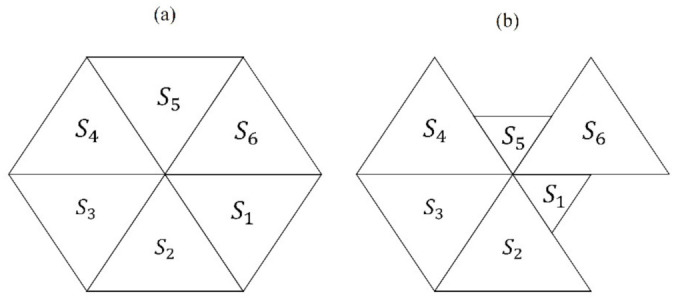
Schematic properties of two different examples of a particular shape Γ. (**a**) A regular hexagon is a partition associated with six sub-areas from six sub-localities $S_{1},S_{2},\ldots ,\;S_{6}$ which are all equal. Then it is non heterogeneous. (**b**) A shape Γ with a six-fold heterogeneous partition such that the areas defined by sub-localities $S_{1}$ and $\;S_{5}$ are smaller than those of $S_{2},\;\;S_{3},\;S_{4}$, and $S_{6}$, then this is heterogeneous.

Partitions $P_{i}$ are sets of areas where each partition is constituted by a subset of a given number $N_{i}$ of sub-localities, $S_{i1},S_{i2},\ldots ,S_{i_{Ni}}$ such that $P_{i}=\cup_{j=1}^{N_{i}}S_{ij}$, where $P_{i}$ is a spatial region which could be either a set of areas as numerical values or any convex polygon in ${\mathbb{R}}^{2}$. In order to start with a statistical description, let $A_{ij}$ be the area of each sub-locality. If $A_{ij}=A_{ik}\forall \;j,k$, then we said that $P_{i}$ is non heterogeneous. In contrast, if exists some j≠k such that $A_{ij}\neq A_{ik}$ then we say that $P_{i}$ is heterogeneous. Therefore, let $A_{i}=\overset{N_{i}}{\underset{j=1}{\sum }}A_{ij}$ be the sum of all of the associated areas of a partition; this set determines a shape $\Gamma =\left\{A_{i}\right\}$. We consider a polygon as a first example of a particular shape Γ with a set of sub-areas which are considered in numerical terms. In that example, the numerical values correspond to triangle areas from a hexagon representing a particular case of a shape Γ (Figure 2).

Therefore, the area average of a partition $P_{i}$ is:

(1) $${\bar{A}}_{i}=\frac{1}{N_{i}}\overset{N_{i}}{\underset{j=1}{\sum }}A_{ij}$$

and

(2) $$\sigma_{i}=\sqrt{\frac{1}{N_{i}-1}\overset{N_{i}}{\underset{j=1}{\sum }}{\left(A_{ij}-\bar{A_{i}}\right)}^{2}}$$

is the standard deviation of each partition. Notice that if $\sigma_{i}=0\;\Rightarrow A_{ij}=A_{ik}\;\forall \;j,k$. Therefore, that partition is non heterogeneous.

Equation (3) reflects the amount of heterogeneity in a given shape Γ and is inside a scale from 0 to ~1. Generalization for equations defining heterogeneity of polygons is written $x_{i}$, where sub-index i reflects the number of sides of each polygon.

(3) $$x_{i}=\sigma_{i}/{\bar{A}}_{i}$$

**Figure 3:**
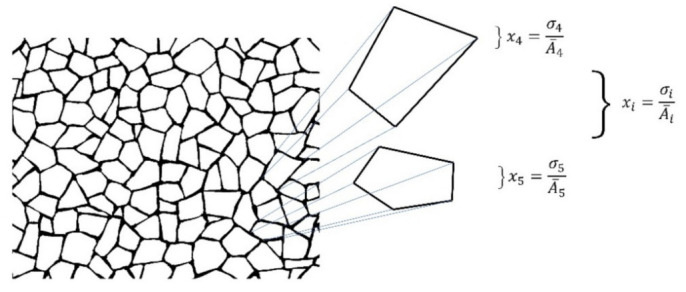
Symbology of equations for individual polygons extracted from a mesh. The expression given by (3) is used in order to obtain individual polygon heterogeneity. Also, the distribution of spatial heterogeneity derived from frequencies levels of heterogeneity in meshes of polygons of BIO, Non-BIO, and RA was defined using the values given by their heterogeneity.

The main objective of our research here is the establishment of a measure of spatial organization which is able to determine the geometrical entropy for biological and non-biological organizations. Therefore, we must relate (3) with a proper collection of shapes Γ reflecting numerical data of spatial heterogeneity in PSP, quantifying indexes of heterogeneity in all of our samples (those from Section 2.2.1). Before defining entropy in mosaics of cells we have to develop a proper methodology to get the coordinates of individual polygons. As mentioned in Section 2.2.1, we used biological (natural) and non-biological processed images (from web sites and references) to define the coordinates of polygons using the centroid of each polygon as the origin of polygonal coordinates. The heterogeneity of each polygon in mosaics was derived with (1), (2), and (3), see Figure 3. With this data and the statistical description of polygons as Γ shapes, we get frequency distributions of heterogeneities for each mesh.

We relate (3) with a proper collection of data reflecting rates of spatial heterogeneity, then quantifying indexes of heterogeneity in all of our theoretical samples. Consequently, our results will be the standard deviation of heterogeneity derived from levels of variability in a collection of shapes Γ from a set of PSP samples. In order to define the standard deviation of heterogeneity we have to determine first the average of PSP heterogeneity for all samples with (4),

(4) $$\bar{x}=\frac{1}{N_{s}}\sum_{s=1}^{N_{s}}x_{is}$$

now, the first subscript *i* of $x_{is}$ correspond to the number of sub-areas, s is the index of a shape, and $N_{s}$ is the total of shapes Γ in a mesh of polygons. The standard deviation can be obtained with (5),

(5) $$\sigma =\sqrt{\frac{1}{N_{s}-1}\sum_{s=1}^{N_{s}}{\left(x_{is}-\bar{x}\right)}^{2}}$$

Equation (5) reflects a global statistical value aiming to determine area variability and the informational entropy.

## 3. Results

### 3.1. Continuous Distribution of Heterogeneity for Shapes Γ-PDA

**Figure 4:**
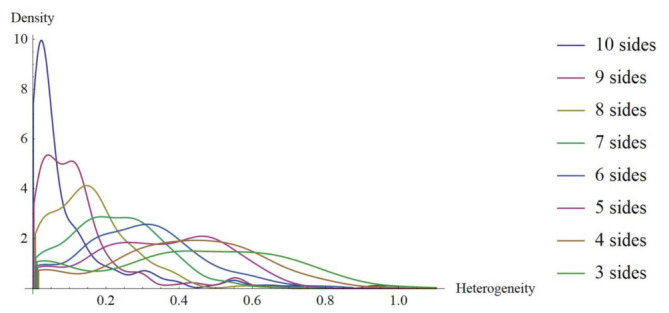
Probability density function for distributions of Γ-PDA applied to transform histograms with discrete values (modified from [16]) into continuous graphics. The horizontal axis shows heterogeneity levels derived from Equation (3).

An important question is whether the variable side number of polygonal shapes in the context of heterogeneity might lead to a continual progression in terms of informational amount or not (that is, entropy as a function of polygonal side number). Since sampled meshes (PSP) are sets of mixed polygons with different number of sides this question must be approached using frequency distributions of heterogeneity in the first case of shapes Γ using planar discrete areas inside a disc *(*Γ-PDA) with a fixed number of sides as the independent variable (algorithm and methodology are provided in Appendix A). The main aim for all of these data is whether statistical variations of spatial distributions in polygons have particular attributes to obtain some clues of biological configurations. Initially, we have discrete distributions for heterogeneity data extracted from shapes Γ-PDA, which will be transformed into continuous ones applying the probability density function algorithm (Wolfram Mathematica 9.0; Champaign, IL 61820-7237, USA. Figure 4).

**Figure 5:**
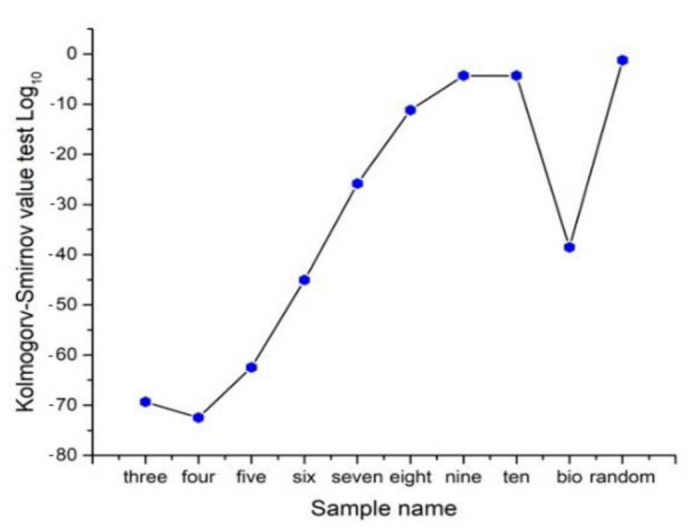
Contrasting continuous distributions of frequencies using heterogeneity data. In order to start with a continuous approach to detect levels of entropy we use Kolmogorov–Smirnov test as a parameter to detect distribution differences between normal distributions and the remaining ones. Heterogeneity values of random sample have the closest value to normal distribution. According to the Log base 10 Kolmogorov–Smirnov test values, partition number four has the lowest values of entropy in continuous terms.

In order to start with a continuous approach to infer levels of entropy, we decided to use a Kolmogorov-Smirnov test between normal distribution of a Gaussian random variable of heterogeneity and the remaining ones to detect distribution differences. For continuous distributions, the maximum entropy corresponds to normal distribution, since a Gaussian random variable has the largest entropy amongst all random variables [52,53]. Therefore, we consider that Kolmogorov–Smirnov test will give us a good proxy of closeness with normal distribution as a first hint of high entropy. According to Figure 5, the Log base 10 of *p*-values of a Kolmogorov–Smirnov test is applied in Wolfram Mathematica 9.0, resulting in a proper comparative of entropy in continuous terms. Frequency distributions of three, four, five, and six Γ-PDA are the four lowest values which is an indicative of low entropy and dissimilarity with normal distribution. In that sense, *p*-value is positively related to entropy, low *p*-values indicate low entropy, high *p*-values indicate high entropy and normality. Kolmogorov–Smirnov test performs the Kolmogorov–Smirnov goodness-of-fit test with null hypothesis $H_{0}$ that data was drawn from a population with a normal distribution and alternative hypothesis $H_{a}$ that it was not (Wolfram Mathematica software 9.0). Also, as an initial experiment one BIO sample (PSD) [40] and one random sample are included, showing that BIO sample has an important contrast with random sample in terms of Kolmogorov–Smirnov test results. The BIO sample reach a value of −38.54 while the random value is −1.23. The two local minima (four-side and BIO samples) are indicative of small *p*-values which reflect that both configurations come from samples with low entropy that is hypothetically frequent in biological arrangements [1].

### 3.2. Bin Categorizations for Measuring Discrete and Continuous Entropy Using Polygons

The Shannon entropy is a parameter indicating a degree of information approaching a resolution of uncertainty. Our description model satisfies the fact that we may work either with frequencies of numerical variables which are sub-areas of polygonal shapes in the context of PSP or with areas using Γ-PDA. Shannon elucidates the convenience of the use of a logarithmic function in the definition of entropy, mainly due to the fact that it is more suitable mathematically since many operations in terms of the logarithm are simpler than in terms of the statistical behavior (the number of possibilities or frequency). In fact, one of our main source ideas is the finding of a practical procedure to retrieve PSP given a geometric informational entropy value. The average surprise of a variable X, which has a distribution $p\left(X\right)$, is called the entropy of $p\left(X\right)$ and is represented as $H\left(X\right)$. For convenience, we often speak of the entropy of the variable X even though (strictly speaking) entropy refers to the distribution $p\left(X\right)$ of X [53]. Thus, the entropy of the heterogeneity variable $x_{i}$ from (3) can be derived from the general formula for discrete values:

(6) $$H\left(X\right)\approx \frac{1}{N_{j}}\sum_{j=1}^{N_{j}}log\frac{1}{p{\left(x_{i}\right)}_{j}}$$

where the subscript j represents the variable number.

**Figure 6:**
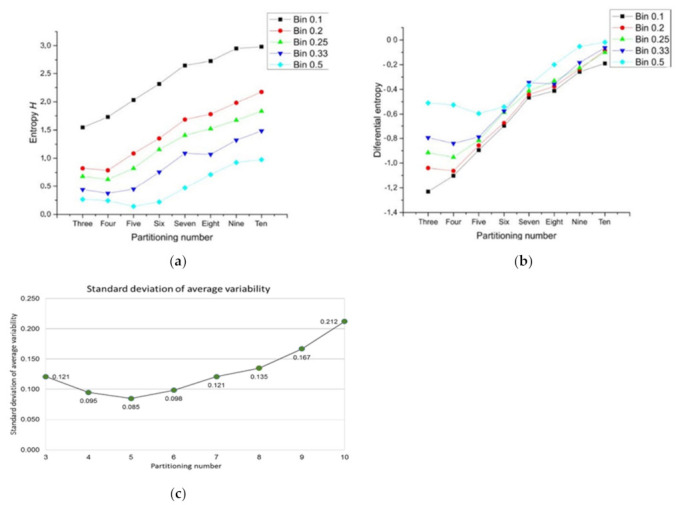
Entropy for Γ-PDA datasets. (**a**) Partitioning number and their associated entropy can be derived from different bin statistical discrete categorization. Graphic shows five bin widths and their associated entropy. Bin width 0.5 has the lowest values of entropy for every partitioning number, meanwhile bin 0.1 statistical categorization has an approximately linear incremental behavior in contrast with the remaining categorizations. In addition, this graphic also shows that there is a similar pattern between discrete and standard deviation of variability (**c**) in terms of the distance from zero using Bin 0.5. (**b**) The associated differential entropy of a partitioning number was derived from Equation (8). Differential entropy datasets show that negative entropy goes from −0.0181 to −1.2309. (**c**) The graphic shows the standard deviation of raw heterogeneity for Γ -PDA using the logarithm base 10, using Equations (4) and (5).

The choice of a logarithmic base regards for a proper election of a unit for measuring information. In consonance with this last idea, we consider frequency values of heterogeneity in a range of bin width. The entropy values using different bin widths (0.1, 0.2, 0.25, 0.33, and 0.5) show that this variable gives an important difference in terms of the decrease of values (Figure 6a from black to green) even in terms of a statistical correlation with raw heterogeneity data (Table 1; for discrete and differential entropy). Regarding differential entropy as a continuous technique, we can consider a formal approximation using:

(7) $$H_{dif}\left(X^{\Delta }\right)\approx \left[\sum_{i}P_{i}log\frac{1}{P_{i}}\right]-\log\frac{1}{\Delta x}$$

where *i* is a subscript referring for the *i*th bin and Δx is the bin width. The count of the *i*th bin is *n_i_* whose area is $a_{i}=n_{i}\times \Delta x$. The total area is $A=\underset{i}{\sum }a_{i}$, and the proportion $P_{i}=a_{i}/A$. Equation (7) derives from:

(8) $$H_{dif}\left(X\right)=\int_{x=-\infty }^{\infty }p\left(x\right)log\frac{1}{p\left(x\right)}dx$$

which is a measure of entropy called differential entropy (continuous entropy) of a variable. Equation (8) can be suited to ignore infinity, so (7) can be derived from (8). For completeness, we measure differential entropy with the data of each sub-areas number considering the five bin width values for discrete Γ-PDA datasets, see Figure 6b (from black to green). Even though each value of a continuous variable can, in principle, convey infinite information, the amount of information it conveys in practice depends on the accuracy of our measurements. In effect, measurement noise divides up the range of a continuous variable into a finite number of discrete intervals; the number of intervals increases as the measurement noise decreases. Therefore, bin width 0.5 represents the noisiest interval of our samples and bin width 0.1 the least of all [53]. Finally, the total standard deviation for discrete entropy values is 0.109905 and for differential entropy is 0.083602. In addition, the total range for discrete entropy goes from 0 to 3, in contrast with differential entropy which reach −1.2 from 0.

### 3.3. Statistical Frequency Distributions of Internal Partition in Γ-PDA and Binary Localities in Bio, Non-Bio, and RA Samples

**Figure 7:**
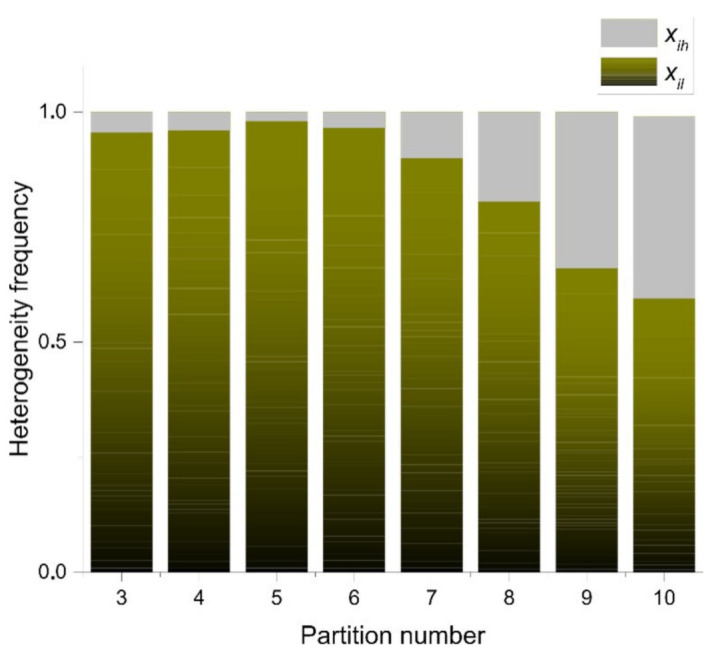
Levels of heterogeneity according to the binary categorization $x_{il}$ and $x_{ih}$. Grey zones are frequency values associated to $x_{il}$ and the green ones are associated with $x_{ih}$. The highest level of homogeneity is for partition number five (grey area), even though, three, four, and six have similar levels. The highest level of heterogeneity is for partitioning number ten.

In order to start with a proper analysis of entropy, we must consider the correlation values of Table 2. Higher correlation values imply a first hint for bin correlation. In spite to seem a weak statistical argument to detect both, the closest bin category and the right mathematical variable to use (either discrete or continuous); this correlation remains variable considering all bin categories. According to partition number the statistical frequency distribution of heterogeneity of Γ-PDA is showed in Figure 7. The bar area determines levels of heterogeneity conforming the binary categorization $x_{il}$ and $x_{ih}$ as a first pragmatic approach. The green area shows levels of high heterogeneity where $0.5\leq x_{i}\;<\;1\Rightarrow X_{i}=x_{ih}$. On the other side, low levels of heterogeneity correspond to the grey area where $0\leq x_{i}\;<\;0.5\Rightarrow X_{i}=x_{il}$, with $x_{il}$ values derived from Equation (3).

**Figure 8:**
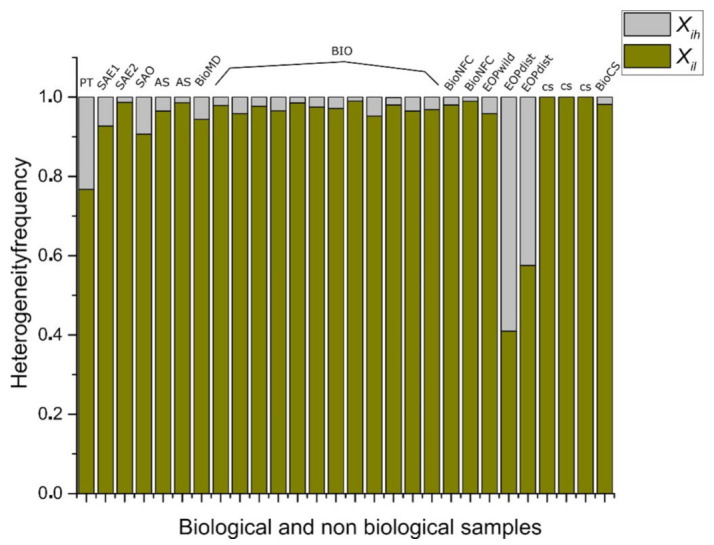
Twenty-eight samples of biological and non-biological simulations of organizations of cells aggregates have a constant high proportion of homogeneity in terms of spatial distribution of inner areas (for name samples and nomenclature of BIO and non-BIO see Table 1). Data from columns BioNFC (Namibia fairy circles), EOP wild (non-disturbed ecological oak pattern) and EOPdist (disturbed ecological oak pattern) shows that at ecological level a wild zone has less heterogeneity polygons that a disturbed zone. The last four samples are biological simulations [20]. The first three simulations result with an entropy of 0. All of these samples result from a dynamical configuration derived from a fine tuning of biophysical parameter variation (line tension and tension values). Even this is happening just when the impairment of the cell division when tension value threshold reaches a 40 percentage with cell proliferation and heterogeneous reduction of line tension among the tissue cells the informational entropy increases up to 0.132065 (BIO CS sample). The first column represents a Poisson–Voronoi tessellation which was used as control.

In order to link entropy and raw geometrical information, our evidence suggests that five-folding organization depicts a sort of spatial organization with low values of information (besides three, four, and six folding organizations, which are also frequent in nature). In fact, this sort of arrangement shows the highest correlation with raw low spatial heterogeneity data for both, differential and discrete entropy (Table 2). As we were pointing out before we consider that binarity must give us some clues in order to understand heterogeneity and discrete entropy (Figure 7) from a simple perspective. The fact that five-fold partitions reflect the most equal distribution of internal space in contrast with other partitions, it is a non-trivial result since this is not a function derived from the polygonal number of sides (Figure 6a,b). According to Figure 8, such as five-fold arrangement, biological organizations of cell aggregates that were derived directly from natural images, or even biological simulations, have a constant high proportion of low heterogeneity in terms of spatial distribution. That implies a clear high degree of homogeneity lying on that proportion of low heterogeneity that is found in all samples derived from biological approximations.

**Figure 9:**
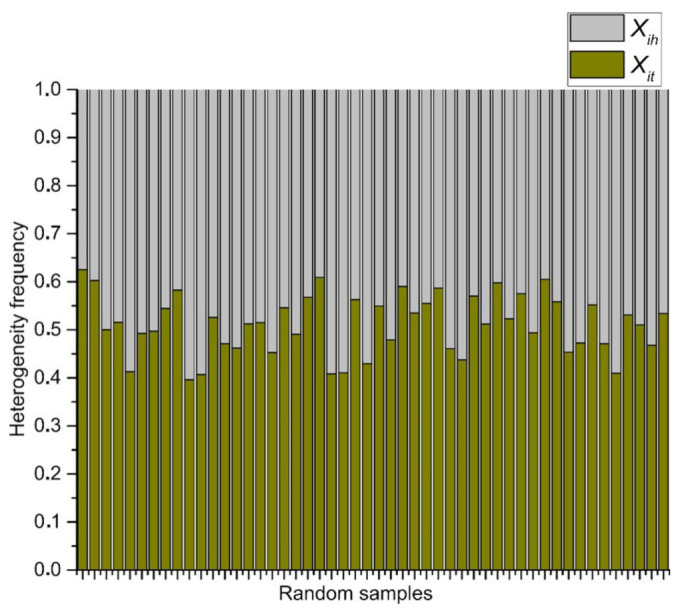
Random arrangements of cells and their heterogeneity frequency. Data shows that random aggregates have an average of an almost half proportion of low heterogeneity (blue) of spatial distribution on internal areas in polygons, and a half of spatial high heterogeneity (grey).

This last result shows the simplicity of approaching the geometry of biological organizations focusing on the binarization data in order to (may) see the main facts of the organizational nature of biological geometries that are often found. In addition, Figure 8 indicates that binarizations works well as an indicator to realize heterogeneity levels in complex meshes of polygonal arrangements since we can see the statistical behavior of data, a high degree of low heterogeneity (homogeneity) and a very low degree of heterogeneity. Random samples are used as a control experiment to visualize contrast in terms of those distributions in Figure 9.

### 3.4. Discrete Entropy for Shapes Γ from Bio, Non-Bio, and RA Samples Using Binarization

**Figure 10:**
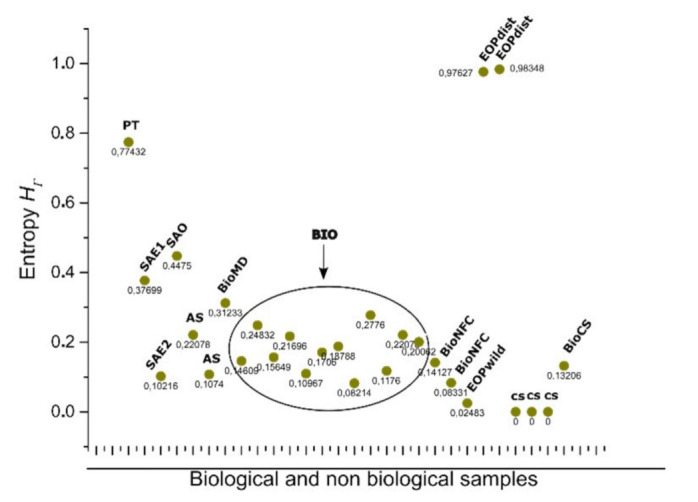
The entropy of cell aggregates groups: biological collected images (BIO; descriptions in Table 1) and processed images which we named non BIO extracted directly from online open access figures; control simulation (CS), simulation at equilibrium (1 and 2) following four interactions of Lloyd’s algorithm (SAE), atrophy simulation (AS), simulation out of equilibrium (SOE), muscular dystrophy (BioMD), and Poisson–Voronoi tessellation (PT). The most abundant area (ellipse) includes BIO data, which is close in terms of entropy with AS, SAE, and SOE. BioNFC (Namibia fairy circles) and EOPwild (ecological oak pattern wild) are also defined by a low degree of entropy. That is not the case for EOPdist (ecological oak pattern disturbed). Control simulation of biological organizations reaches a 0 entropy value. That value can change when biophysical manipulation of parameters is included [20].

In terms of discrete entropy, there are an important number of simulations extracted from the open access figures that are excluded from the BIO zone despite of being considered as simulations of biological samples (Figure 10). All of those collected simulations were based on quantified distances from SDCP conserved in proliferating metazoans tissues with a polygonal frequency of 49% six-sided polygons, 29% five-sided polygons, and 20% seven-sided polygons, which is traditionally used as reference in epithelial studies. Control simulation (CS), simulation at equilibrium (SAE), atrophy simulation (AS), and simulation out of equilibrium (SOE) were computational simulations of cells aggregates assuming variations as metric distances from Lewis’s Law values or holders of another kind of biological or physical properties. The Shannon entropy associated with biological simulations, such as CS reaches levels of zero entropy, which implies null information which is not the case for either Γ-PDA associated with hypothetical biological morphologies or samples constricted to the BIO zone. However, there was a sample that increase their entropy according to some simulation conditions (Figure 10). Certainly, Poisson–Voronoi tessellation (PT) was used as control since we assumed that its arrangement would be far away from the order zone. Hence, the geometry between BIO and non-BIO arrangements of internal space are underlying important differences whose consequences and effects would define particular behavior in actual biological organizations.

As we can see at Figure 10 the BIO zone includes images that are not being simulated, such as dWP, dWL, and BCA (Table 1). However, simulations that have some kind of manipulation can increase their heterogeneity resulting in highest entropy than control simulations without parameter variation. One interesting point comes from the ecological oak patterns which are disturbed and non disturbed oak zones [48]. The level of entropy increases whether the zone is a perturbed ecosystem or not.

**Figure 11:**
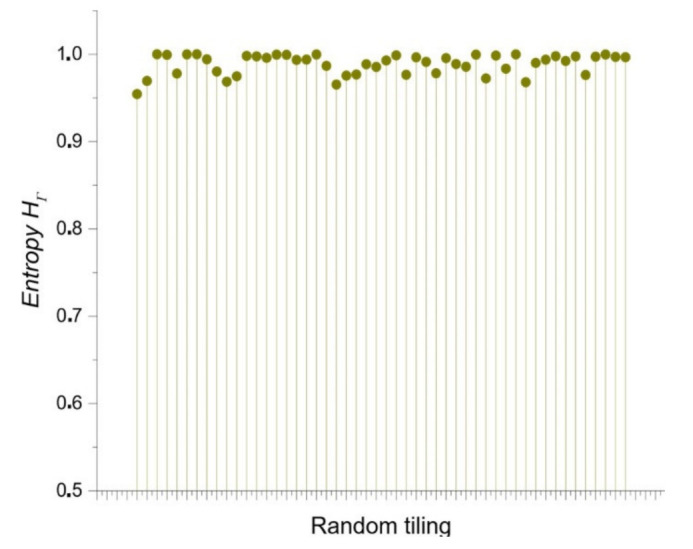
The discrete entropy of random samples (dataset derived from Figure 9). The entropy values are almost constantly in line with maximum entropy.

The Shannon entropy associated with RA frequency distribution (Figure 9) is an expected result, whose discrete entropy values are around 1 (Figure 11).

### 3.5. Continuous Entropy for Shapes Γ from Bio, Non-Bio, and RA Samples

**Figure 12:**
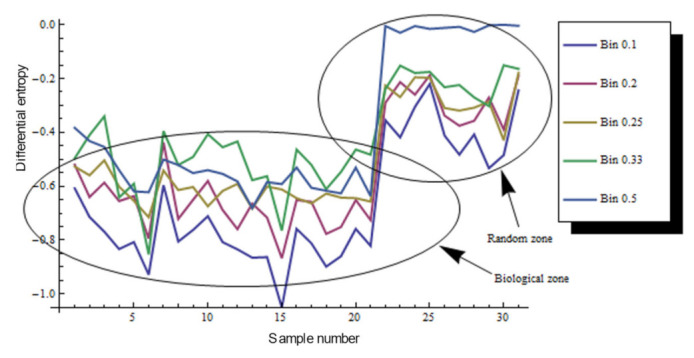
Differential entropy for total datasets. The continuous approach reflects that entropy values are negative in 21 Bio samples and the results are related Bio, non-Bio (SOE, SAE, and AS) and RA samples.

To estimate the entropy of any variable, it is necessary to know the probability associated with each of its possible values [53]. As we point out (Section 3.1) probability density function is a well-accepted starting reference to estimate a continuous distribution from discrete possible values. In fact, it has been an important mathematical trouble that has been solved arriving to Equation (8). In addition, Equation (8) can be useful even with discrete values using bin areas $a_{i}$. In order to establish a panoramic view of continuous entropy values we consider getting the values from Bio, non-Bio (SOE, AS, and SAE) and RA samples. According to Figure 12 there are two negative intervals for all bin categories.

Despite being working with the same data we have an important gap among discrete entropy and differential entropy (Figure 6a,b) given that the first results are positive and the second negative. Regarding this last point, we will develop some more hypotheses at a later time. 

## 4. Discussion

We may see that three, four, five, and six-fold Γ shapes as planar discrete areas (Γ-PDA) behave as almost egalitarians in terms of raw spatial inner heterogeneity (Figure 6c) which we consider as a first reference of geometrical constraint in biological organizations. Beside this last fact, we consider as an important issue to be the differential entropy derived from the geometry of polygonal shape patterns (PSP) samples whose values remain close to those of Γ-PDA (Figure 6b). In addition, both partitioning number of shapes Γ-PDA and their associated entropy and the differential entropy derived from the geometry of PSP can be derived from different bin statistical discrete categorization. Figure 6a,b show five bin categorizations and their associated entropy (bin width 0.1, 0.2, 0.25, 0.33, and 0.5) for discrete and continuous values. Given that bin width 0.5 has the highest correlation with raw spatial heterogeneity for both values of entropy (Table 1), we decided to use it as the main dataset to observe discrete entropy at Section 3.3. On the other side, bin width 0.1 statistical categorization has a linear incremental behavior in contrast with the remaining categorizations. According to the elected binary system, where low heterogeneity is in the range 0 < = $X_{il}$ < 0.5 and high heterogeneity is in the range of 0.5 < =$\;X_{ih}$ < = 1, biotypical arrangements distributes internal space in a very egalitarian statistical way. For discrete values an interval of entropy values emerges, clustering arrangements from biological samples (around 0.08 and 0.27 bits of entropy; Figure 10). Section 3.3 shows the methodology to analyze discrete entropy using three types of mosaics (PSP): Random arrangements (RA), natural images extracted from the web (BIO), and processed images also extracted from the web (Non-BIO): which we named control simulation (CS), simulation at equilibrium (SAE), atrophy simulation (AS), simulation out of equilibrium (SOE), and Poisson-Voronoi tessellation (PT). Spatial heterogeneity in mosaics of polygons was derived using (3) for each polygon and discrete entropy using (6). Random arrangements of cells and their heterogeneity frequency shows that random polygonal aggregates representing cell aggregates have an average of an almost half proportion of heterogeneity of spatial distribution on internal areas in polygons with a nearby equal half of spatial homogeneity (Figure 9). In fact, this result explains by itself how is that highly heterogeneous partitions gives a highly entropic result.

Biological simulations (which we have included both as part of non BIO samples) of organizations of cells aggregates have a constant high proportion of homogeneity in terms of spatial distribution of inner areas. Some other approaches have found similar results, such as that analyzing avian photoreceptor patterns representing a disordered hyperuniform solution to a multiscale packing problem [54]. In fact, the penultimate three samples (CS) areas in biological simulations assuming Lewis’s Law have a 100% degree of homogeneity (Figure 8). Then, a high degree of homogeneity in a computational simulation following some algorithmic instructions could derive in a beautiful representation following the SDCP of a real biological sample but a considerable lack of substantive geometric information. Thus, levels of intrinsic disorder (heterogeneity) emerging from the actual biological forms are necessary to have a proper simulation. A typical statistical approach using just statistical differences between different polygonal organizations shall not integrate this last key issue. Despite found statistical variations between BIO and Non-BIO organizations for PSP in terms of discrete entropy, differential entropy shows a better resolution (with an $\bar{\sigma }$ of 0.115982 in contrast with $\bar{\sigma }$ of 0.187632 for discrete values) resulting in an interesting gap for all bin categorizations ($\bar{X}=-0.61872$). To finish with the discussion about the continuous subject, we shall remark that this research is not inside the interdisciplinary field of information geometry. Despite this, there are some interesting methodological convergences that can be visited at [55,56]. In addition, we considered that the main convergence lay on a very interesting epistemological subject, geometry as a source of information. On the other hand, regarding discrete entropy, BIO group is between 0.08 and 0.27 bits which is a range for entropy values including three, four, five, and six folding partitions which are very common in nature. Also, in Figure 8 and Figure 10 the first value represents a Poisson–Voronoi tessellation (PT) which was used as a control since this mesh is derived from a well know non-ordered organization of points. Even this sort of organization is not biological it seems not be inside the gap of random organizations for discrete entropy. The most abundant grey area of Figure 10 is considered as the BIO zone, which also include AS (that is a non-Bio sample). Hence, the atrophy of some simulations increases their heterogeneity degree which finally derives in a biological-like outcome. Regarding the differential entropy the Bio zone is a clear interval showed at Figure 12 which remains with a notable distance from random differential entropy. In that sense, considering the continuous approach where the inclusion of Non-Bio into BIO group seems clear is not an unexpected result since computational simulations representing algorithmic instructions are perturbed in a way that could easily derive in a biological entropy position. It does not happen with control simulations since heterogeneity does not appear at all. Hence, the algorithmic constructions showed on this paper are following hidden mathematical prescriptions reveling high levels of homogeneity beside another fundamental nature of the BIO group, a lightly bias disruption of order. In fact, five control simulation group whose main feature has been the closeness with SDCP (CS right side) have values of zero entropy (Figure 8 and Figure 10).

On the other hand, MD seems to be a close object to BIO realm. However, it is not inside the limits. We consider that it is an important find since our parametric measure of geometric information can give us some clues about pathological routes in a very simple way, that important finding agrees with [43]. At the level of ecological scales, we include just two image samples that were very representative. Namibia fairy circles are one of the most interesting results since we confirmed some previous hypothesis about the potential of free scale approaches to understand biological organizations [46].

## 5. Conclusions

The main goal of this research lies on the intriguing question whether geometry is an actual source of information defining biological arrangements. The Shannon information of an outcome is also called surprisal since it reflects the amount of surprise when that outcome is observed [53]. In the context of information theory, the fact of being surprised requires knowing which outcomes are more surprising and which are less surprising. According to this last idea, we have specific statistical distribution of spatial heterogeneity frequencies for Bio, Non-Bio, RA, and Γ-PDA using collections of individual polygons and disc simulations. All of these outcome frequencies are treated as outcome probabilities that are giving us particular levels of discrete and differential entropy for biological organizations using pure geometry. High levels of heterogeneity imply an intrinsic amount of surprise in contrast with a high degree of heterogeneity using the binarization approach. Therefore, our results reflect that there is a potential informational limit for biological organizations in terms of discrete and differential entropy. Despite of the value of this result there is still a broad distance to conclude that the differential entropy interval represents a unique range since it is not the same for discrete entropy. A deep mathematical and computational research is still lacking in order to define the limits of biological geometric information of polygonal aggregates. However, biological organizations are complex spatial systems which should be constrained into a narrow window of variability depending on levels of heterogeneity that can be translated into informational entropy. Paradoxically, we can see a myriad of morphological variations in nature. We conclude that the statistical properties of biological architectures can be manifested into an overwhelming number of morphologies since all of them are singular possibilities in a realm of pure organization with particular geometrical attributes (such as heterogeneity). In that sense, shape is a constant dynamical composition of arrangements and an opening infinite possibility of configurations with spatial confined attributes as a consequence of its essential organization which depends on their own informational limits. According to our results, we consider that homogeneity with very low levels of heterogeneity in biological systems is a fundamental factor for biological organizations (e.g., network theory calls it sparsity). Hypothetically, in the context of complex adaptive systems spatial heterogeneity could be associated with a source of variation (or noise) and degrees of freedom, which is notably a different perspective from the pure blueprint genetic approach, whose information lies exclusively onto molecular and ontogenetical basis. With this in mind, we consider that the value and limits of informational entropy for geometrical systems in biology is a novelty approach with a potentially width domain of impact.

## Figures and Tables

**Table 1:** Summary of category, abbreviation, particular name, and number of samples.

Mesh Categories	Abbreviation	Name and Number of Samples
-	PSP	Polygonal shape pattern (total number of samples 38)
-	Γ-PDA	Planar discrete areas (8)
Bio	dWP	Drosophila prepupal wing discs (3)
Bio	dWL	Middle third instar wing discs (4)
Bio	BCA	Normal human biceps (2)
Bio	MD	Muscular dystrophy from skeletal muscles (1)
Bio	PSD	Pseudo stratified Drosophila disk epithelium (4)
Bio	NFC	Namibia fairy circles (2)
Bio	EOP	Ecological Oak Patterns (3)
Non-Bio	CS	Control simulations (5)
Non-Bio	SOE	Simulation out of equilibrium (1)
Non-Bio	SAE	Simulation at equilibrium (2)
Non-Bio	AS	Atrophy simulation (2)
Non-Bio	PT	Poisson–Voronoi tessellation (1)
RA	RA	Random arrangements (50)

**Table 2:** Correlation values between discrete and differential entropy with standard deviation of heterogeneity raw data.

Bin Width	r between Dis_E and STD_HRD	r between Dif_E and STD_HRD
0.1	0.7215	0.7405
0.2	0.8129	0.8191
0.25	0.8161	0.8221
0.333	0.8642	0.8667
0.5	0.9311	0.9308

Dif_E = differential entropy; Dis_E = discrete entropy; r = correlation; STD_HRD = standard deviation of heterogeneity raw data.

**Figure A1:**
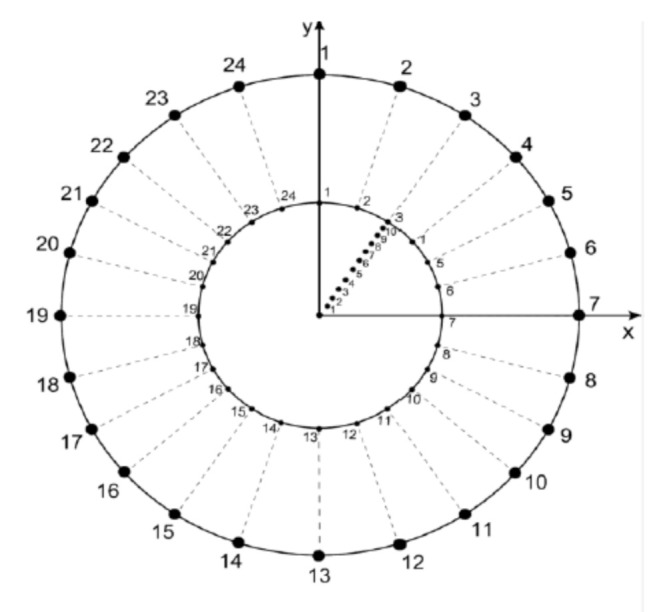
Defining partitioning number and partition variability. A disc is constructed to get Voronoi diagrams with constant area despite variability. The magnitude of the radius defines ten levels of partition variability: (a) 1, (b) 1-2, (c) 1-3, (d) 1-4, (e) 1-5, (f) 1-6, (g) 1-7, (h) 1-8, (i) 1-9, and (j) 1-10. Each level of variability is given by radii ranges except (a) which is fixed at 1.

**Figure A2:**
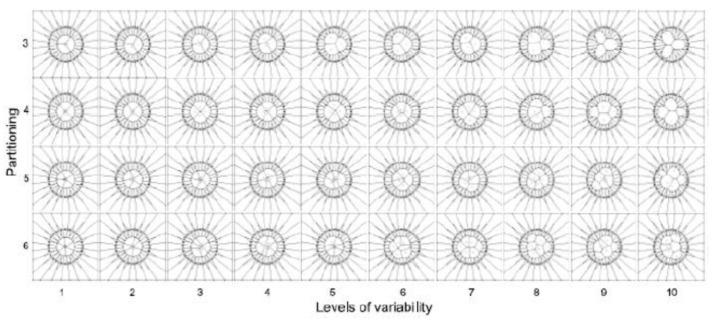
Partitioning number and partition variation of planar discs. A sample of 40 planar discs shows how partitioning number (vertical left side) determines segmentation of an almost constant area (≈108.5 ± 1.5) into a particular number of sub-localities. Partition variability (bottom horizontal numbers) installs levels of variability giving 10 constant and subtle increases of area to generate random segmentations using Voronoi tessellations.

## Data Availability

The sources of the data used in this study are mentioned throughout the paper.

## Appendix A. A Numerical Approach Using Partitions of Shapes Γ-PDA (Planar Discrete Areas)

A complete view of a wide spectrum of planar discrete areas (PDA) is obtained if we design a numerical model. Our geometrical design has as a first condition, namely the fact that shapes Γ-PDA with different number of sub-localities remains with a constant area during the experiment in preparation for obtaining normalized data. In order to establish variability inside a constant area, we consider two conditions for shapes Γ-PDA: (a) they must remain with an almost constant area during the experiment where partition $P_{i}$ range from 3 to 10 sub-localities (eight categories); and (b) also each partition $P_{i}$ must include 10 levels of variability. Therefore, each partition $P_{i}$ with a particular constant area has 10 levels of variability during the experiment. We must be aware that shapes Γ-PDA is a particular case of a partition $P_{i}$.

For this purpose, we use Voronoi diagrams to model space of shapes Γ-PDA with different number of parts (from 3 to 10) where two variables are studied, namely partitioning number (pn) and partition variability (pv), which are defined as follows:

a. The partitioning number (pn) defines the number of partitions inside a disc (ranging from 3 to 10): Each partition $P_{i}$ is constituted by a subset of a given number $N_{i}$ of sub-localities, $S_{i1},\;S_{i2},\ldots ,S_{iN_{i}}$ such that $P_{i}=\cup_{j=1}^{N_{i}}S_{ij}$, where $P_{i}$ is a spatial region which could be any Γ -PDA in ${\mathbb{R}}^{2}$.
b. Partition variability (pv) determines multiple levels of variability (10) inside each pn by using random points, which in turn will define the Voronoi diagrams.

The algorithm to build pn and pv is described in the next seven steps as follows:

1. Features of the external disc: the boundaries of the external limit are defined by 24 fixed points generated as follows: The radius of the external disk is set to *r =* 1 and consecutive points are separated by an angle *θ*/24 (where *θ* corresponds to 2π). Point 1 is aligned with axis *y* (Figure A1).
2. Features of the internal disc: the boundaries of the internal limit are defined by 24 fixed points generated as follows: The radius of the internal disk is initially set to *r =* 0.53 ± 0.4 with 24 points consecutively separated by an angle *θ*/24. Point 1 is aligned with axis *y*. (Figure A1).
3. Partitioning number (pn): once the number of partitions is defined, say *n* (where 3 ≤ *n* ≤ 10 and n∈ℤ), points are located in the disk at angles 2π/*n* ± 0.069 radians but at different radius. These radius values will define the pv, as described in the next item.
4. Partition variability (pv). For each angular region defined above, 10 points are located at radius (between r = 0 and r = 10) at different positions to define different degrees of variability (diagonal points of internal disc at Figure A1). The first point (first level of variability) is at r = 1. After the second point, all of them are located at random radius between 1 to 10. Hence, each level of variability (10) is given by radii ranges except 1 which is fixed at 1 (diagonal points of internal disc); (a) 0 to 1, (b) 0 to 2, (c) 0 to 3, (d) 0 to 4, (e) 0 to 5, (f) 0 to 6, (g) 0 to 7, (h) 0 to 8, (i) 0 to 9 and (j) 0 to 10.
5. Voronoi tessellations: the partition variability will define the broad spectrum of possibilities for area distribution inside discs without losing partitioning number using Voronoi tessellations.
6. Area average: according to Equation (1), the average of areas requires a summation of sub-localities areas $\left(A_{ij}\right)$ which were derived from pn with a changing variability pv.
7. Data mining: once the partition areas $\left(A_{ij}\right)$ inside discs were obtained and (1) was solved, (2) is used to obtain standard deviations $\left(\sigma_{i}\right)$ of variability for each disc. In order to normalize the level of variability for each pn, an index dividing the standard deviation of partitions and the particular area average of each partition was obtained (variability average; Figure A2). There are eight particular area averages of partitions since we have a sample of 8 discs with different pn (from 3 to 10). These particular area averages are derived from a value *n*/(≈108.5 ± 1.5) which are *n* values obtained from the first level of variability (pv) at r = 1. It is important to say that the radius of the external disc (1) and the radius of the internal disc (*r* = 0.53 ± 0.4) was modified in order to get the particular area averages. However, despite the modification, the index between external discs and the internal ones remains constant. A sample of 20 discs to get 20 standard deviations was generated for each pn, and for each level of pv (10) giving a sample of 200 discs for each pn. An average of standard deviations (${\bar{\sigma }}_{i}$; variability average) was derived for each level of variability.
8. Standard deviation. Finally, a standard deviation of all variability averages is obtained for each pn.

Table A1 shows the area at internal disc, and the area average, for particular partition numbers.

**Table A1:** Level of variability and area average according to the partition number.

Partition Number	Area at Internal Disc (Level of Variability Pv1)	Particular Area Average
3	107.2	35.7354
4	108.7	27.1963
5	109.5	21.9155
6	109.9	18.3248
7	110.1	15.74
8	110.32	13.7959
9	110.51	12.2794
10	110.605	11.0605
